# Wiggler radiation at a low-emittance storage ring and its usage for X-ray absorption spectroscopy

**DOI:** 10.1107/S1600577521012844

**Published:** 2022-01-18

**Authors:** Konstantin Klementiev, Hamed Tarawneh, Pedro Fernandes Tavares

**Affiliations:** a MAX IV Laboratory, Lund University, PO Box 118, SE-221 00 Lund, Sweden

**Keywords:** wiggler, low-emittance storage ring, EXAFS

## Abstract

A wiggler is a high-power insertion device that was used in the past to produce a smooth wide-band X-ray spectrum. It is widely believed that on low-emittance synchrotrons this X-ray source loses its spatial and spectral homogeneity and therefore becomes less ideal than a scanning undulator. In this paper, we report on experimental and computational studies of an in-vacuum wiggler installed on the first fourth-generation synchrotron MAX IV. We investigate how several physical parameters affect the wiggler spectrum and propose a combination of a few of them that results in significant spectral smoothing. We also examine EXAFS spectra for possible distortions originating from the source imperfection. For this purpose, we scrutinize samples of various homogeneity. We conclude that wigglers are still an appropriate class of insertion devices, also on low-emittance synchrotrons.

## Abstract 2

Experimental and computational studies of wigglers on low-emittance storage rings are reported. EXAFS spectra for possible distortions originating from the source imperfection are also examined. It is concluded that wigglers are still an appropriate class of insertion devices, also on low-emittance synchrotrons.

## 1. Introduction

Undulators generate X-rays with the spectral distribution consisting of narrow-band harmonics. To obtain a harmonic at a specific energy, the undulator device has to be properly tuned by arranging its magnet girders at a given spacing — the magnetic gap. The motion speed of these many-ton girders becomes a serious limiting factor in quick energy scanning applications, such as scanning X-ray absorption spectroscopy. For quick energy changes, wide-band sources seem more appropriate. Wigglers — high flux wide-band sources — were popular on second-generation synchrotrons, less so on third-generation machines, and almost absent on (the existing or planned) fourth-generation storage rings. Two primary reasons for the latter are (i) a high heat load on the beamline optics and (ii) possible spatial and spectral non-uniformity that may affect absorption spectra (Welter, 2012 ▸; Klementiev *et al.*, 2016 ▸). In the energy domain, this non-uniformity is seen as a ripple of a few percent amplitude, which consists of a sequence of many undulator harmonics still not fully merged although the undulator deflection parameter *K* is big, *K* ≫ 1. On the older machines, the non-uniformity was typically smeared out (with probably only one exception at the Canadian Light Source, see below), while on the latest fourth-generation storage rings it becomes visible.

One standard way to smoothen the spectrum is to gradually vary the magnetic field by tapering the magnetic gap. This approach was also adopted at MAX IV’s Balder beamline. At commissioning, the first energy scans revealed that the amplitude of the ripples was significantly smaller and with another beating pattern compared with calculations, at that time made without magnetic field errors. It was realized afterwards that the taper effect interfered with the shift of the observation direction — technically achieved by vertically tilting the electron beam. Those observations made us search a wide parameter space for optimal settings that deliver X-ray absorption spectra of the highest quality.

Wiggler spectra were earlier computationally studied by Walker (1998 ▸, 2003 ▸) and Bahrdt (2016 ▸). At low energies, the spectrum has well separated undulator harmonics. At higher energies, the interference effects become smaller and the spectrum eventually becomes smooth and similar to that of a bending magnet. Walker also lists the factors that help to smooth out the spectrum: wavelength acceptance, angular acceptance, electron beam divergence, electron beam energy spread and magnetic field errors. A typical monochromator bandpass is too small to have a significant effect; the most dominant is the angular acceptance, followed by electron beam divergence (emittance) and energy spread and magnetic field errors (Walker, 1998 ▸, 2003 ▸). Off-axis wiggler radiation also attains spectrum smoothing and this effect has an analogy with magnetic field tapering (Walker, 1988 ▸). However, the interplay between off-axis tilting and magnetic field tapering was not investigated.

To our knowledge, there are no published systematic studies, neither computational nor experimental, of the above smoothing effects. The HXMA wiggler beamline at the Canadian Light Source has implemented some magnetic field randomization. In combination with a widened angular acceptance, this measure has resulted in a smoothened wiggler spectrum, which was briefly reported by Jiang *et al.* (2007 ▸).

In this paper, we investigate the dependence of the wiggler spectrum on tapering and off-axis vertical pointing angle (tilting). As will be shown, there are other influential factors, such as electron beam emittance (with a surprisingly small effect), imperfections in the wiggler magnets and electron beam energy spread. We also compare the experimental wiggler spectra with calculated ones, which makes us believe that all relevant factors are accounted for and understood. In our calculations, we could afford not to drop any of these factors, which represents some improvement compared with the studies by Walker (1998 ▸, 2003 ▸) (without energy spread effects in the calculations) and by Bahrdt (2016 ▸) (with energy spread but without emittance and finite beamline acceptance).

The final goal of all these exercises is to improve the quality of the resulting absorption spectra. As will be shown, the quality is not only affected by the inhomogeneity of the X-ray beam in energy and real space but also depends on sample quality.

The Balder beamline is the first wiggler beamline on fourth-generation storage rings and has been in official user operation since September 2019. This paper summarizes our commissioning and operational experience from a wiggler beamline on a fourth-generation storage ring. The work should be relevant for several synchrotron facilities worldwide that consider future upgrades of their accelerator complexes towards diffraction-limited light sources.

## 2. Balder beamline

Balder is a wiggler beamline dedicated to X-ray absorption spectroscopy (XAS) and X-ray emission spectroscopy (XES) in the tender and hard X-ray energy range 2.4–45 keV (Klementiev *et al.*, 2016 ▸). We aim to reach a high repetition rate down to 1 s for full EXAFS to preserve the sample (reduce radiation damage by shortening exposure time) and attain redox dynamics in *in situ* reactions.

The beamline is situated at the bigger 3 GeV ring of the MAX IV Laboratory in Lund, Sweden. The 2 m-long in-vacuum wiggler (Marcouille *et al.*, 2010 ▸) with *K*_max_ ≃ 8.5 feeds the beamline with X-rays of a maximum accepted power *ca* 1 kW through the front-end masks of maximum 400 µrad (h) × 100 µrad (v) angular size. The total power radiated by the insertion device is 18 kW at the smallest magnetic gap of 4.5 mm and the nominal ring current 500 mA. The studies reported here were performed at a ring current of 120–140 mA and beamline acceptance of 250 µrad (h) × 25 µrad (v) being a typical acceptance value in user experiments.

The optics consists of a vertically collimating mirror, a cryogenically cooled double-crystal monochromator (DCM) and a toroidal focusing mirror that focalizes the beam down to a ∼70 µm × 70 µm spot. The DCM is of a direct-drive motorization type, *i.e.* without reduction gears, allowing a scanning speed up to 5° s^−1^. During energy scans, the parallelism of the crystals is dynamically maintained by a piezo-driven fine-pitch stage controlled by an MoCo device developed and produced by ESRF (ESRF, 2003 ▸). The MoCo is fed by two signals (top and bottom) from a split-collector ionization chamber *I*_0_ situated upstream of the sample and keeps the two signals equal during energy scans. This stabilization scheme — and not the elsewhere frequently used *intensity* stabilization — appears very handy at long energy scans when the *I*_0_ signals change significantly. Long energy scans were extensively used in the present work. It is worth noting here that the tilting variations in the source (see below) were followed by a quick tuning procedure comprising a rocking curve scan and a pitch scan of the focusing mirror, so that the MoCo was always fed by two approximately equal, and also optimal, signals.

## 3. Experimental

**Figure 1:**
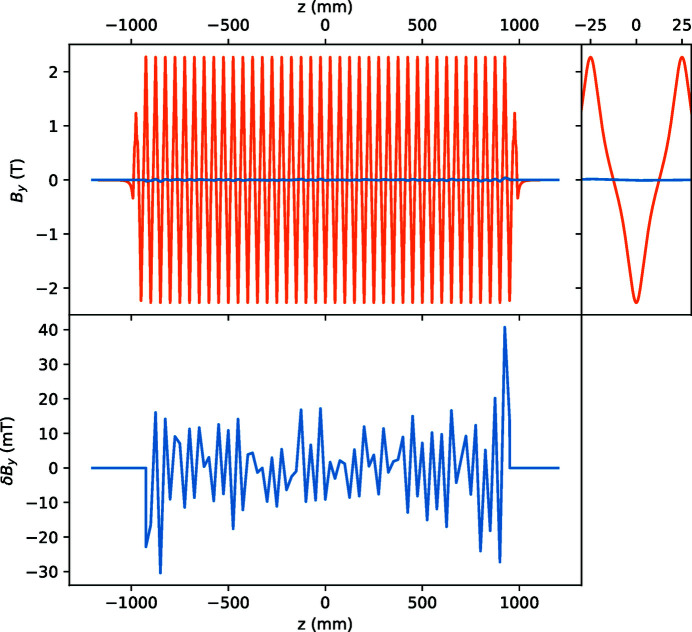
The vertical magnetic field of IVW50. Orange: ideal calculated field, also zoomed in the small right-hand panel. Blue in the top and bottom panels: measured deviation from the ideal field.

The magnetic field map used in the source simulation was based on magnetic measurement of the peak field in all periods using the Hall probe bench at Synchrotron SOLEIL (Marcouille *et al.*, 2019 ▸). The measured field deviation values, see Fig. 1 ▸, were then used to scale the simulated magnetic field for each half-period. The wiggler was first commissioned in the 3 GeV ring in February 2017 to characterize its impact on the electron beam in terms of orbit distortion, beta beat and emittance damping, as shown by Tarawneh *et al.* (2019 ▸).

The energy scans were obtained in the continuous scanning mode (also known as fly mode) when the two involved motors — the Bragg axis and the perpendicular translation of the second crystal — are continuously moving with the measurements being triggered at predefined Bragg angles. The scanning speed was set to moderate and resulted in ∼100 s duration of a 4–9.5 keV scan. The lower energy range limit was set to 4 keV, not to the beamline’s limit of 2.4 keV, for a practical reason: one gas filling of the *I*_0_ ionization chamber can serve the whole energy range if it starts at a reasonably high energy. The higher limit was motivated by the fact that at energies higher than 9 keV the undulator features (ripples) become practically negligible. This energy range, covering the *K*-edges of 3*d* elements and the *L*-edges of the rare earths, is highly demanded by the user community of the beamline and has been requested by *ca* 60% of all accepted proposals at Balder (MAX IV, 2021 ▸).

The beamline does not have control over the electron beam parameters: shift and inclination in the wiggler’s straight section, emittance and energy spread. These were changed on demand by the storage ring operator during a few *ad hoc* commissioning shifts spread over several months. The intensity detectors (ionization chambers) were not identically set during these experimental sessions, which explains some variation in the plotted signal level in different figures.

The tilt range was limited to ±100 µrad, as advised by the accelerator group. This range is comparable with the natural divergence of synchrotron light: 1/γ ≃ 170 µrad for a 3 GeV machine. By comparing the negative and the positive tilts, a small shift of the tilt origin of ∼−10 µrad was detected and ascribed to the mechanical alignment precision of the front-end elements. Below, we show only the negative branch in order to unclutter the figures. We also remain at the formal definition of the tilt, as defined by the e-beam position monitors.

To verify the mechanical precision of the insertion device motorization, the magnetic gap taper was scanned from zero to the maximum (2 mm over 2 m length) and then backward. The obtained dependencies were indistinguishable for the two directions and only one direction is shown below.

The extended X-ray absorption fine-structure (EXAFS) spectra at the Fe *K*-edge were measured in transmission detection in the continuous scanning mode. Each spectrum of 1.2 keV range took *ca* 1 min.

Two powder pellets were taken from the stock of beamline standards, one denoted as ‘good’ (hematite) and one denoted as ‘bad’ (iron sulfate). The difference is in the resulting grain size, which depends on the material hardness and the time spent for grinding. A good sample should have grains smaller than the absorption length — a few µm at ∼7 keV. On the contrary, a bad sample has large grains partially acting as beam stops with pinholes in between letting the beam through without absorption. In both cases, the total powder mass was optimal for transmission detection, as calculated by the *XAFSmass* code (Klementiev & Chernikov, 2016 ▸).

## 4. Computational

The calculations of the radiated field from an insertion device with a real (including distortions and tapering) magnetic field is a heavy computational problem because the calculations have to be made over the full length of the insertion device, not just over one magnetic period. Moreover, for large observation angles in the case of a wiggler, much larger than for an undulator, the convergence is more difficult to achieve, which requires a dense calculation mesh along the electron trajectory. Non-zero energy spread in the electron beam adds yet another dimension to the problem.

Here, we used the code *xrt* (Klementiev & Chernikov, 2014*b* ▸) to calculate the wiggler radiation in the full-length undulator approach. The code calculates an optimum mesh partitioning that guarantees convergence also in the difficult cases of large observation angles and high photon energies. The code was validated against other popular codes: *SRW* (Chubar & Elleaume, 1998 ▸) and *Spectra* (Tanaka & Kitamura, 2001 ▸). The comparison tests are available on the *xrt* documentation pages (Klementiev & Chernikov, 2014*a* ▸). In addition to the optimized convergence, another advantage of *xrt* is the ability to use the high computational power of GPUs on workstations or computing clusters. The storage ring parameters used for the calculations are summarized in Table 1 ▸.

In order to compare the calculated flux with the measured current from the *I*_0_ ionization chamber, several material factors were applied: transmittivity of filters, reflectivity of two mirrors and two crystals, absorption by the ionization chamber gas mixture and the conversion factor photon energy to electron–ion pair number. The latter was taken from the ‘Orange’ X-Ray Data Booklet (CXRO & ALS, 2009 ▸), while the other factors were calculated by *xrt*.

## 5. Studies of the wiggler beam

**Figure 2:**
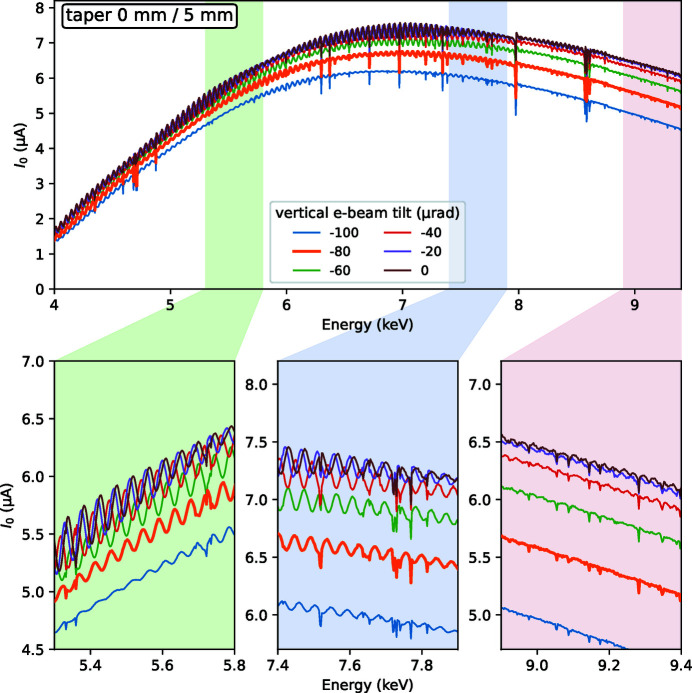
Experimental wiggler spectra measured as intensity upstream of the sample (*I*_0_) during quick energy scans at various e-beam inclination and taper values of the magnetic gap. The typical operational inclination value is −80 µrad, shown by a thicker orange line. An animated version of the figure can be found in the supporting information.

The measured wiggler spectra at various taper and e-beam tilt values are shown in Fig. 2 ▸. Three typical energy ranges zoomed in the lower part of the figure are part of EXAFS regions at the Ti, Fe and Cu *K*-absorption edges. The following experimental features are worth noting:

(i) The spectra have oscillations in energy with period 45–50 eV at the 5 mm gap. With larger gaps, the period elongates and the amplitude becomes bigger (not shown).

(ii) The spectra have sharp, ∼1 eV wide, intensity drops, so-called monochromator glitches. At these particular Bragg angles, multiple Bragg conditions are fulfilled at the monochromator crystals and the beam is diffracted elsewhere, not only towards the working direction, thus reducing the useful flux.

(iii) Upon increasing the gap taper, the *I*_0_ oscillations attain a beating pattern with a decreasing period.

(iv) When inclining the electron beam, and thus effectively changing the observation direction, the intensity decreases and the harmonic oscillations become smaller.

**Figure 3:**
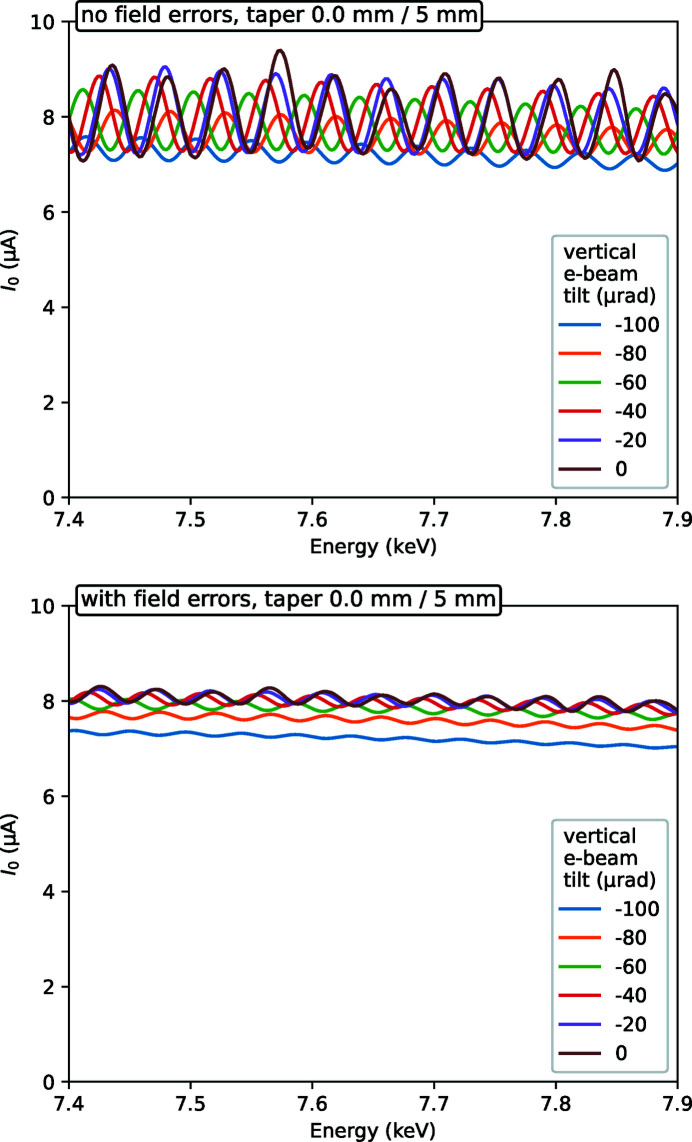
Calculated wiggler spectra at various e-beam inclination and taper values of the magnetic gap. Top: with the ideal magnetic field; bottom: with the magnetic field corrected for the measured field errors. An animated version of the figure can be found in the supporting information.

To understand all influencing factors onto the *I*_0_ oscillations, we tried to reproduce them by calculations, see Fig. 3 ▸. The calculations correctly reproduce the oscillation frequency and the overall dependence on e-beam inclination and magnetic gap taper. One important result of the calculations is that the present field errors strongly reduce the oscillation amplitude at zero taper, *cf*. the first animation frames of the top (no errors) and bottom (with errors) panels in Fig. 3 ▸. On the other hand, the taper represents a stronger effect on the oscillation amplitude than the present field errors, and even the first step in gap tapering (0.1 mm over the wiggler length) reduces the oscillations by almost an order of magnitude for the ideal wiggler case. The calculated beating pattern, however, does not fully reproduce the measured curves, primarily because the measured field errors were sampled only up to the frequency of the inverse magnetic period, *i.e.* measured at each magnetic pole, so that higher field harmonics did not receive proper scaling.

**Figure 4:**
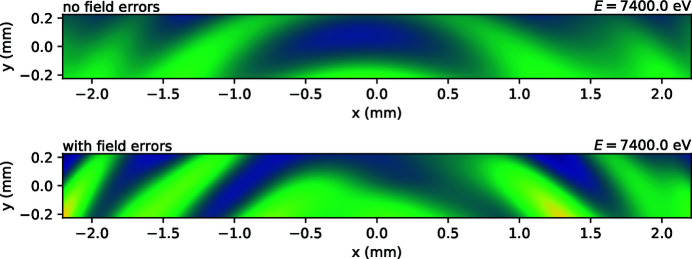
Calculated transverse intensity distribution at the front-end movable mask at *ca* 18 m from the source. The e-beam was tilted by −80 µrad (typical operational tilt) and the taper was set to 0.25 mm (typical operational taper). The field of view corresponds to 250 µrad (h) × 25 µrad (v). The color map range is common for both cases. An animated version of the figure can be found in the supporting information.

The calculations also reveal the transverse distribution in the monochromatic beam, see Fig. 4 ▸. We also tried to experimentally record a similar distribution on a diamond fluorescent screen situated downstream of the DCM, where the beam is almost the largest along its propagation path. Whereas the undulator rings were clearly visible at large magnetic gaps, by decreasing the gap below 10 mm we found no contrast on the apparently uniformly illuminated screen. In the calculated transverse maps in Fig. 4 ▸, the visibility function is <10%, and the visible contrast is obtained by applying a color map. Despite the ‘no field error’ case having somewhat lower contrast, its regular round harmonics generally result in a larger flux variation upon spatial integration over the screen area when energy is scanned over one ripple period, here ∼45.6 eV. A distorted field generally results in a smoother spectrum but still may have local spatial inhomogeneity with a larger amplitude.

**Figure 5:**
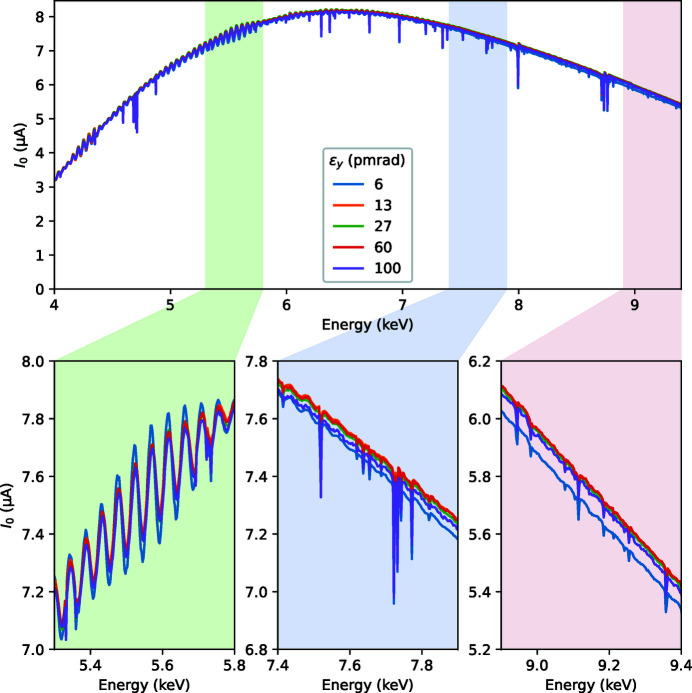
Experimental wiggler spectra at various vertical e-beam emittance ɛ_*y*_, in the presence of field errors, with 0.25 mm taper and −100 µrad tilt.

**Figure 6:**
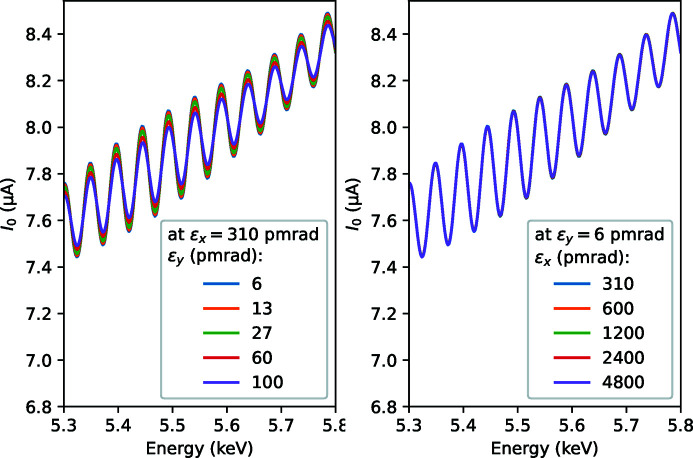
Calculated wiggler spectra at various vertical (left) and horizontal (right) e-beam emittances.

Another parameter to study is electron beam emittance. As a reminder, the undulator ripples in wiggler spectra were typically not visible at older generation synchrotrons. Therefore, emittance was expected to be the main controlling factor. We could experimentally vary the vertical part of it, see Fig. 5 ▸. Quite surprisingly, the effect of this variation is weak, and we could closely reproduce it in calculations, see Fig. 6 ▸, left. Horizontal emittance we could not vary, so its effect was studied only computationally, see Fig. 6 ▸, right. Even more surprisingly, its effect is almost absent. This paradoxical result can be understood by comparing the angular source size with the corresponding angular acceptance. Despite the horizontal emittance being much bigger than the vertical one, the angular source size in the horizontal is only nearly twice as large as the vertical size. On the other hand, the typical horizontal acceptance is ten times bigger than the vertical one, see Fig. 4 ▸ for the aspect ratio. Therefore, the relative convolution effect of the angular source size is weaker in the horizontal, which explains the weaker effect of horizontal emittance.

**Figure 7:**
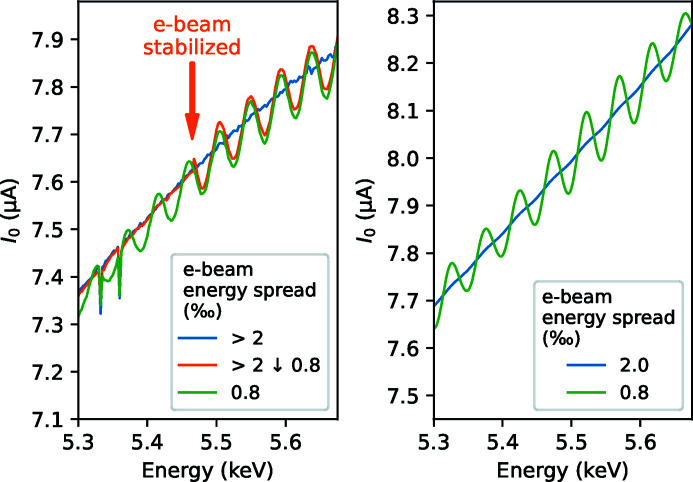
Left: experimental wiggler spectra at various e-beam energy spreads. A large energy spread is not typical and was due to a transient e-beam instability. The stabilization regain was instantaneous and happened during an energy scan (the orange curve). Right: calculated spectra under similar conditions.

If it is not the emittance, then what is the decisive factor that creates ripples in the wiggler spectrum at new storage rings? A part of the answer was found accidentally when the electron beam was temporarily longitudinally unstable. The longitudinal instability was caused by a faulty setting in a low-level RF feedback loop that controls the amplitude and phase of the accelerating voltage in one of the 100 MHz active cavities in the ring (Andersson *et al.*, 2011 ▸). The resulting longitudinal (*i.e.* time-energy) oscillations caused electron energy spread to increase to >2 × 10^−3^ when the wiggler spectrum became flat (blue line in Fig. 7 ▸, left). Once the fault was fixed, the longitudinal instability disappeared, the energy spread was brought towards its nominal value ∼0.8 × 10^−3^ and the undulator ripples were restored, see the orange and green lines in Fig. 7 ▸, left. The energy spread was estimated from the horizontal beam sizes measured at two locations along the ring where the dispersion function is nearly zero and where it is high (Breunlin & Andersson, 2016 ▸).

As with the other effects influencing spectral smoothness, we could closely reproduce the effect of electron energy spread in calculations, Fig. 7 ▸, right.

Finally, it is not small emittance per se that results in pronounced undulator ripples in wiggler spectra at last-generation synchrotrons. Rather, several other factors have gradually evolved: energy spread has become slightly smaller (at least at MAX IV), beamline acceptance is also getting smaller with each generation of storage rings (also partly due to ever-increasing radiation safety requirements), the assembly quality of modern insertion devices has improved, and the overall alignment precision of insertion devices, front-end elements and beamline optics has increased. All these factors lead to increased undulator ripples.

With the optimal settings of the photon source — 0.1 mm to 0.25 mm taper depending on energy range and the maximum allowed e-beam inclination (presently, −80 µrad) — the undulator features can be reduced by approximately an order of magnitude for a reasonably small loss in flux.

## 6. Impact of *I*_0_ variations on EXAFS spectra

If all beamline detectors were ideally linear in intensity, *I*_0_ variations should be present in all measured signals with the same relative strength. In such a case, the normalized signals, *i.e.* after division by *I*_0_, should only have sample-related features — absorption edges, diffraction reflexes *etc*. Inversely, non-linear detectors may result in the presence of *I*_0_ variations in the normalized signals. Another point of concern is non-homogeneous beam intensity that may vary in space during energy scans and thus variously probe the sample. To disentangle detector non-linearity from illumination inhomogeneity, we have measured EXAFS spectra of differently uniform samples by utilizing variously inhomogeneous beam.

We have chosen EXAFS at the Fe *K*-edge (∼7.1 keV) for a few practical reasons. The undulator ripples are still quite noticeable at this energy (decreasing at higher energies) and air absorption is negligible thus allowing a simple experimental setup. Metal foils are the most uniform samples; we used an iron foil of 7.5 µm thickness. Powder samples require some skill for achieving sample uniformity; we measured two samples of different quality, denoted as ‘good’ and ‘bad’.

The undulator ripples were controlled by selecting magnetic gap tapering. We used two taper values, 0 mm and 1 mm, that resulted in a similar ripple amplitude but a slightly shifted weight over the EXAFS energy range, and a value 2 mm that resulted in a larger ripple, *cf*. the animation frames in Fig. 2 ▸ in the supporting information in the middle zoomed plot.

**Figure 8:**
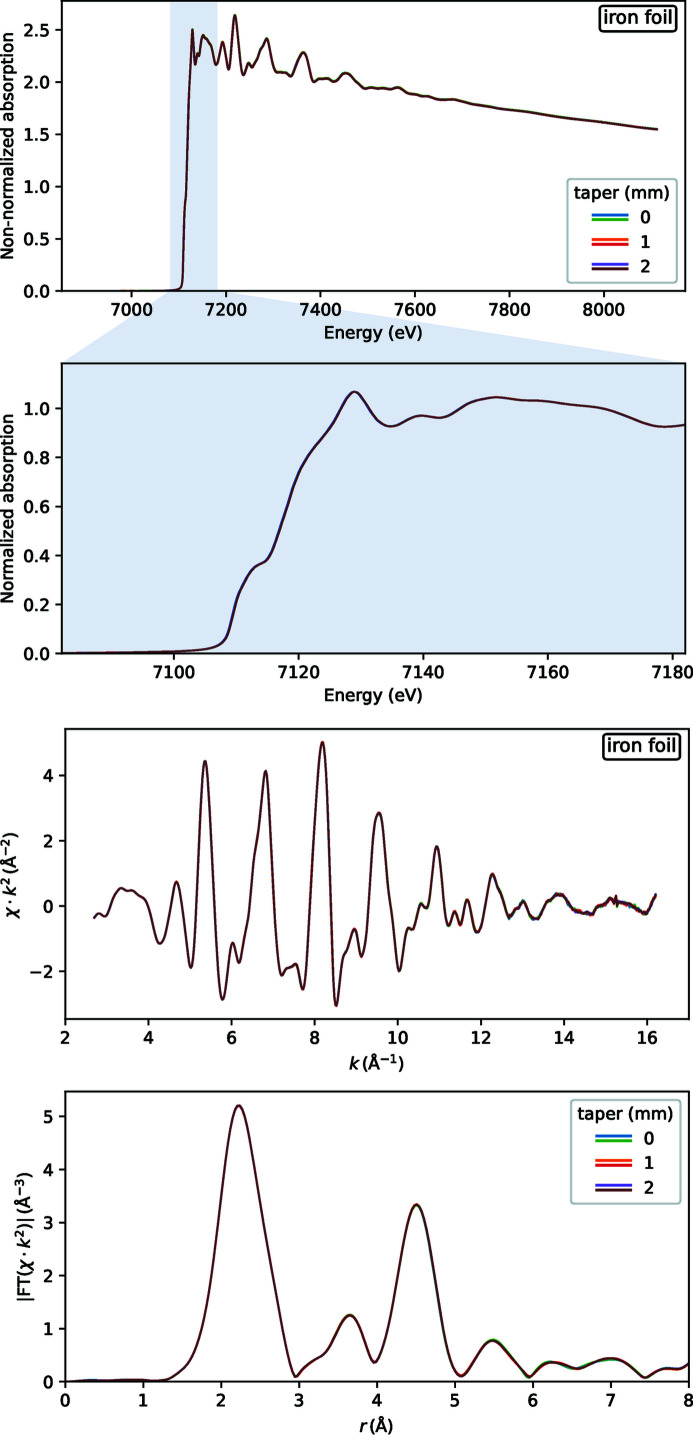
EXAFS spectra for a sample with the most homogeneous thickness: a metal foil. Three values of wiggler gap taper were tested, each with two repeats of EXAFS, six curves in total in each plot.

The EXAFS spectra of the foil are shown in Fig. 8 ▸ in energy, photoelectron wavenumber *k* and phase-uncorrected real space *r*. The six measured spectra fully merge, so the three different ripple patterns do not propagate from the raw measured signals into the EXAFS spectra. This fact proves, in turn, the detection linearity.

**Figure 9:**
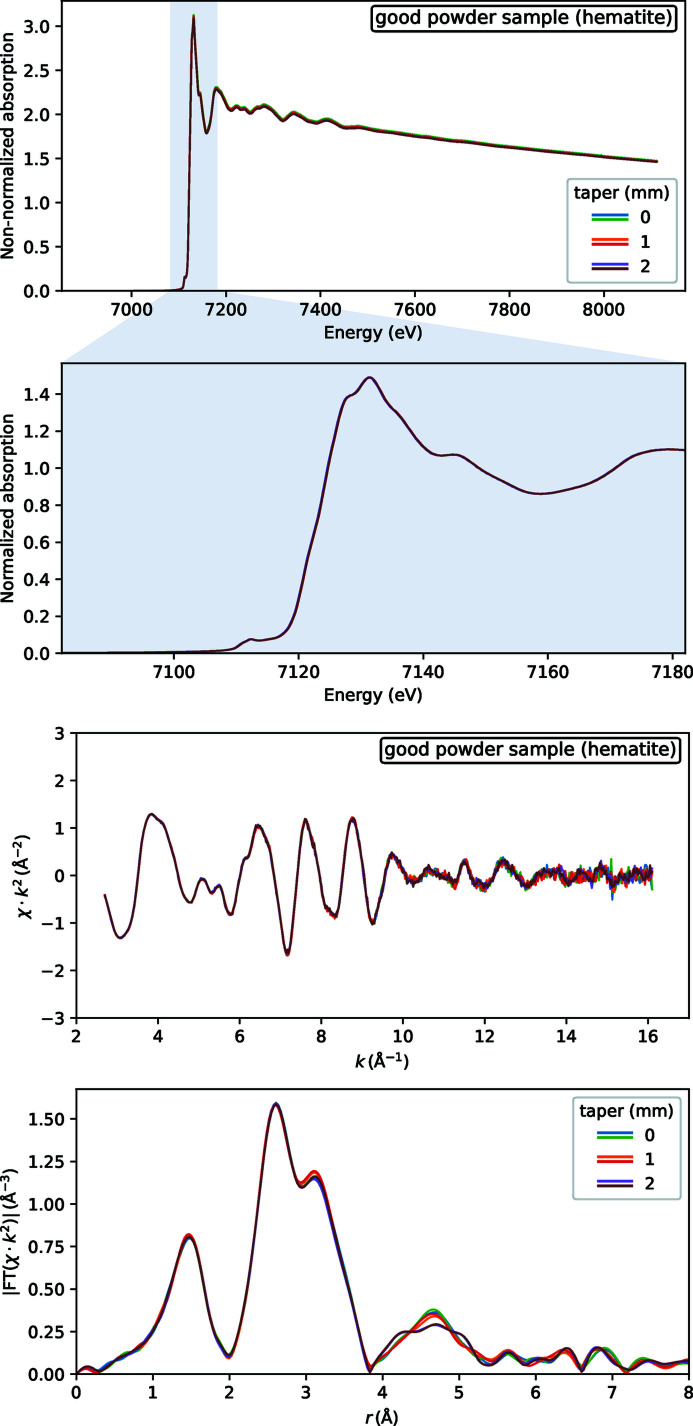
EXAFS spectra for a sample with a fairly homogeneous thickness: a high-quality pressed pellet. Three values of wiggler gap taper were tested, each with two repeats of EXAFS, six curves in total in each plot.

**Figure 10:**
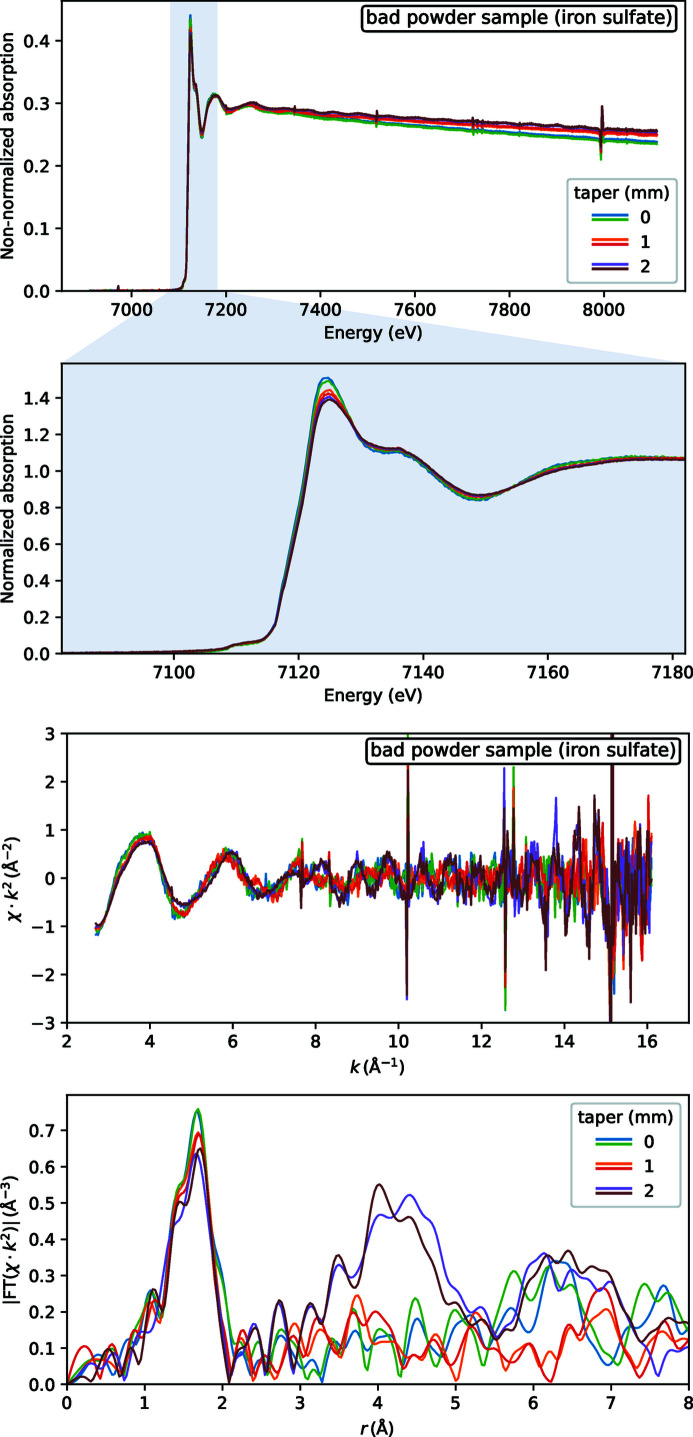
EXAFS spectra for a sample with an inhomogeneous thickness: a low-quality pressed pellet. Three values of wiggler gap taper were tested, each with two repeats of EXAFS, six curves in total in each plot.

The *I*_0_ ripples do propagate into EXAFS spectra of powder samples. This is visible to a much lesser extent for high-quality samples (Fig. 9 ▸) — here only the FT spectra at 4–5 Å (phase uncorrected) have some scatter. Low-quality samples have much stronger distortions: some foreign high-frequency oscillations are obvious in *k*-space EXAFS and even XANES has some contrast losses, see Fig. 10 ▸.

The EXAFS spectra were measured with the beam focalized at the sample position, so that the spatial beam inhomogeneity visible in the original divergent beam, as in Fig. 4 ▸, is mixed back at the sample but probably imperfectly. This may explain the propagation of *I*_0_ oscillations into EXAFS spectra, as the brighter beam regions may meet large particles or otherwise pinholes in a periodic manner during an energy scan.

While undulator ripple may cause severe distortions of EXAFS spectra, another artifact seems to be of similar importance: monochromator glitches. Their propagation into EXAFS is also related to sample homogeneity: non-uniform samples exhibit more glitches in EXAFS. The Balder DCM has many glitches of various strengths. There is one particularly strong glitch at 8.00 keV, see Fig. 2 ▸, that happens at the end of the Fe *K*-edge EXAFS. This glitch is barely visible on the foil spectrum at *k* ≃ 15 Å^−1^ (note that the spectrum is magnified by *k* ^2^), is noticeable on the ‘good’ powder spectrum, and has an enormous destructive influence on the ‘bad’ powder spectrum. We see that even if the undulator ripple could be fully removed, non-uniform samples are hardly usable due to the presence of monochromator glitches. On the contrary, uniform samples are not susceptible to *I*_0_ variations caused by monochromator glitches or undulator ripples.

It is worth noting that this study of *I*_0_ oscillations influencing EXAFS spectra was performed for a non-optimal combination of magnetic taper and electron beam inclination. With the optimal settings — 0.1 mm to 0.25 mm taper and the maximum allowed e-beam inclination (presently, −80 µrad) — the requirements on sample homogeneity are fairly less strict.

## 7. Conclusions

We have studied various factors controlling the smoothness of wiggler spectra on the fourth-generation synchrotron source MAX IV. Among the main factors are electron beam energy spread and the presence of magnetic field errors in the insertion device. Small electron beam emittance — the main characteristic of fourth-generation synchrotrons — has a much weaker effect.

To reduce the undulator harmonics, present as a ripple on the wiggler spectrum, we propose a combination of wiggler gap tapering with electron beam inclination. Future wiggler beamlines may yet improve the smoothness by introducing controlled magnetic field perturbations during the design phase of their insertion devices, as was first done at the Canadian Light Source (Jiang *et al.*, 2007 ▸).

The undulator ripple has an oscillation period of a few tens of eV and may resemble EXAFS oscillations. Whether this ripple propagates from *I*_0_ into absorption spectra depends on sample homogeneity. This contamination of EXAFS, should it happen, is also accompanied by monochromator glitches. The latter may serve as an indicator and their absence may assure that EXAFS is clean from false oscillation frequencies.

Finally, this study has shown that wigglers may serve high-speed spectroscopy beamlines also at low-emittance synchrotrons. The inherent spectral ripple does not compromise EXAFS quality provided the samples are of sufficient thickness uniformity.

## Supplementary Material

Animated version of Figure 2. DOI: 10.1107/S1600577521012844/hf5425sup1.pdf

Animated version of Figure 3. DOI: 10.1107/S1600577521012844/hf5425sup2.pdf

Animated version of Figure 4. DOI: 10.1107/S1600577521012844/hf5425sup3.pdf

## Figures and Tables

**Table 1:** Parameters of the storage ring used in the calculations

Parameter	Value	Unit
Energy†	3	GeV
Current‡	120–140	mA
Energy spread†	8.3 × 10^−4^	
Horizontal emittance†	310	pm rad
Vertical emittance‡	5.5	pm rad
Horizontal betatron function†	9.539	m
Vertical betatron function†	1.982	m

† Nominal value.

‡ As measured.
