# Supplementary material for: Wiggler radiation at a low-emittance storage ring and its usage for X-ray absorption spectroscopy
Source: J Synchrotron Radiat. 2022 Jan 18;29(Pt 2):462–9. doi: 10.1107/S1600577521012844 (PMC8900845; doi:10.1107/S1600577521012844)
Supplement: Supplementary file 1 [file s-29-00462-sup1.pdf]

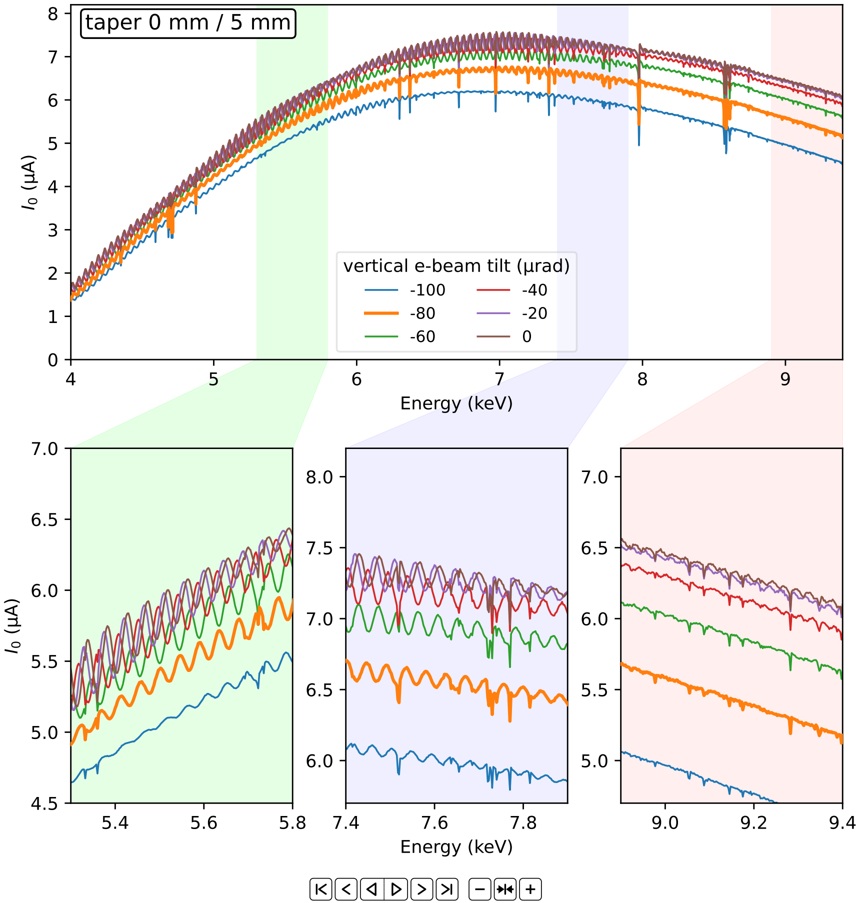

Figure 2

(7 animated frames) Experimental wiggler spectra measured as intensity upstream of the sample (I_0) during quick energy scans at various e-beam inclination and taper values of the magnetic gap. The typical operational inclination value is −80 µrad, shown by a thicker orange line.
