# Supplementary material for: Wiggler radiation at a low-emittance storage ring and its usage for X-ray absorption spectroscopy
Source: J Synchrotron Radiat. 2022 Jan 18;29(Pt 2):462–9. doi: 10.1107/S1600577521012844 (PMC8900845; doi:10.1107/S1600577521012844)
Supplement: Supplementary file 2 [file s-29-00462-sup2.pdf]

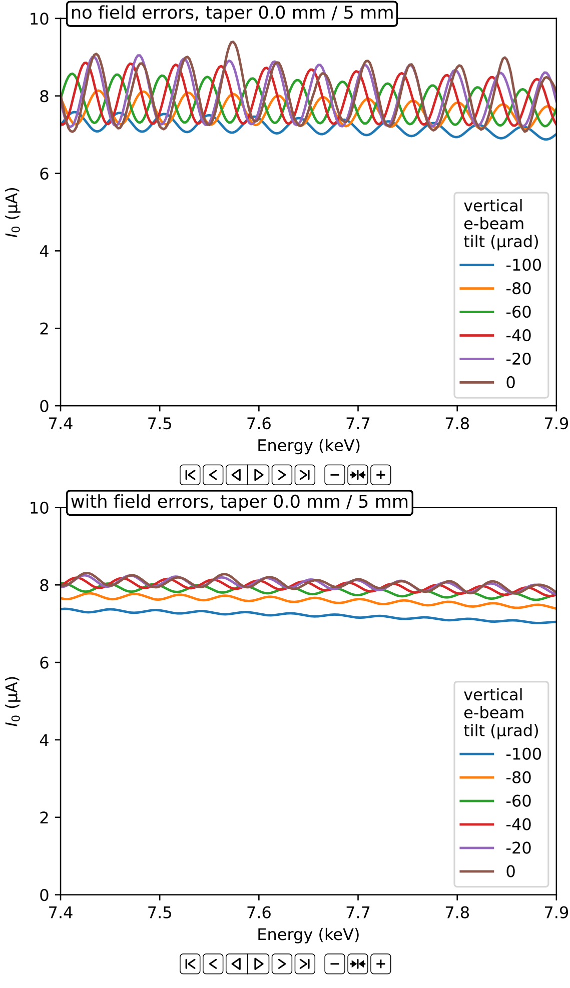

Figure 3

(7 animated frames) Calculated wiggler spectra at various e-beam inclination and taper values of the magnetic gap. Top: with the ideal magnetic field, bottom: with the magnetic field corrected for the measured field errors.
