# Supplementary material for: Wiggler radiation at a low-emittance storage ring and its usage for X-ray absorption spectroscopy
Source: J Synchrotron Radiat. 2022 Jan 18;29(Pt 2):462–9. doi: 10.1107/S1600577521012844 (PMC8900845; doi:10.1107/S1600577521012844)
Supplement: Supplementary file 3 [file s-29-00462-sup3.pdf]

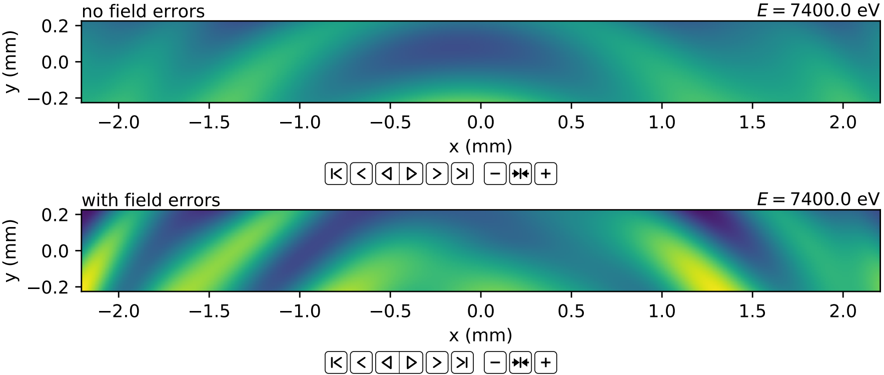

Figure 3

(9 animated frames) Calculated transverse intensity distribution at the front-end movable mask at ca. 18 m from the source. The e-beam was tilted by -80 µrad (typical operational tilt) and the taper was set to 0.25 mm (typical operational taper). The field of view corresponds to 250h × 25v µrad^2. The energy range in this animation is one harmonic period of 45.6 eV. The color map range is common for both cases.
